# Protein aggregates and biomolecular condensates: implications for human health and disease

**DOI:** 10.3389/fmolb.2025.1719678

**Published:** 2025-12-02

**Authors:** Ambuja Navalkar, Anoop Arunagiri, Tovaria Kee, Kathigna Panchal, Kathryn Dick

**Affiliations:** 1 National Centre for Cell Science, Savitribai Phule Pune University Campus, Pune, Maharashtra, India; 2 Department of Biological Sciences, East Tennessee State University, Johnson City, TN, United States

**Keywords:** protein aggregates, biomolecular condensates, protein misfolding, proteinopathies, amyloid

## Abstract

Biomolecular condensates are at the forefront of understanding biological concepts, representing one of the most revolutionary areas in cell biology over the last decade. Numerous proteins, peptides, and nucleic acids have been shown to form membrane-less organelles, also known as condensates, in cells, demonstrating their functional relevance. Multiple research approaches in the fields of physics, chemistry, and biophysics investigate the underlying multivalent interactions that influence the phase separation of biomolecules. As failure to regulate condensate properties, such as formation and/or dissolution has been postulated as a driver of the misfolding and aggregation of proteins in stress, aging, and neurodegeneration disorders, understanding the fundamentals of condensate assembly has been considered of utmost importance. In this review, we will focus on the key regulators and biophysical drivers of phase separation and protein aggregation, evidenced in the literature. We will elaborate on the dynamic interplay between phase separated and aggregated state, highlighting the emergent properties of condensates that can contribute to the misfolding of proteins in the context of physiology and diseases. An in-depth understanding of condensate pathology can reveal novel avenues for targeting proteinopathies linked to misfolding.

## 1 Introduction

A plethora of proteins maintain cell homeostasis, which is facilitated by protein conformations often associated with specific functions. In recent years, research has focused on understanding the biophysical properties associated with protein assemblies and how they relate to function and diseases (Jucker and Walker, 2018; Kroschwald et al., 2022). Amino acid sequence and the environment (solvent milieu) in which the protein is present can dictate the protein folding landscape (Rumbley et al., 2001), in which a vast array of possible conformations a protein can adopt. Within this landscape, the protein structure assumes a specific three-dimensional arrangement by which it can perform its biological function. This structure typically includes primary, secondary, tertiary, and at times quaternary levels of organization (Englander and Mayne, 2014). Protein structural elucidation is important for understanding how proteins fold, function, and interact (Chiesa et al., 2020). Researchers have adopted a range of methods, such as Nuclear Magnetic Resonance (NMR) spectroscopy, X-ray crystallography, and, more recently, AlphaFold, aimed at accurately experimentally determining protein states and predicting structures, respectively (Jumper et al., 2021; Kuh et al., 2019). Proteins achieve their specific native structure through a precise and complex folding process. The formation of partially folded intermediates and the crucial involvement of various cellular components, including molecular chaperones, various ions, and inorganic molecules, can influence protein stability and interactions. In some cases, cellular membranes can provide a scaffold or environment for folding (Saibil, 2013). When one or more of these factors fail to exist or function, the protein folding process can go awry, leading to misfolded structures that deviate from their native conformation (Kha et al., 2022). One such outcome is the formation of amyloids—insoluble fibrillar aggregates characterized by a cross-β-sheet structure. Amyloid formation is often associated with a shift in the protein energy landscape, where misfolded intermediates become trapped in energetically stable states (Kha et al., 2022; Eisenberg and Jucker, 2012). While the amyloid state is not completely understood, the formation of protein aggregates is implicated in a variety of neurodegenerative diseases, including Alzheimer’s, Parkinson’s, and Huntington’s disease (Eisenberg and Jucker, 2012). A subset of protein aggregation, however, is reversible and has been evidenced to be associated with function (Hammer et al., 2008; Iconomidou and Hamodrakas, 2008). Functional amyloids are fascinating examples of how proteins can self-assemble to benefit cells across all life forms (Gebbink et al., 2005; Badtke et al., 2009; Maury, 2009). Unlike the pathological amyloids associated with diseases, these evolved structures perform native-like functions, acting as crucial components in various biological processes (Gebbink et al., 2005; Badtke et al., 2009; Maury, 2009). The precise self-assembly allows them to carry out vital roles within the cell without the cellular toxicity, which is often associated with the pathological amyloids (Iconomidou and Hamodrakas, 2008; Wang and Chapman, 2008). Studying how the protein landscape contributes to both proper functions associated with folding and pathological misfolding provides critical insights into disease mechanisms and opens potential avenues for therapeutic intervention.

In the recent decade, an interesting paradigm shift has been observed in the field of protein assemblies due to the observation of organizational assemblies called biomolecular condensates (Banani et al., 2017; Lyon et al., 2021; Li et al., 2022). With the emerging evidence of biomolecular condensates, the process of phase separation has been extensively recalled as an underlying driving force. In cells, phase separation has been predicted to organize specific biomolecules like proteins, RNA, and DNA into dynamic, membrane-less compartments (Fare et al., 2021). This organization has been speculated to be associated with diverse but targeted cellular roles by proteins (Fare et al., 2021). Phase separation has been postulated to facilitate the formation of various membraneless organelles or compartments within the cytoplasm and nucleus. Few extensively studied examples of biomolecular condensates include: processing bodies (P bodies), stress granules (SGs), Balbiani bodies, germ granules, promyelocytic leukemia (PML) bodies, Cajal bodies, nuclear speckles, and nucleoli, among others (Banani et al., 2017; Fare et al., 2021; Alberti and Hyman, 2021; Li et al., 2022).

Biomolecules within these condensates are shown to be highly dynamic, and the dynamicity has been measured using fluorescence-based techniques like FRAP. However, in the recent decade, several studies have focused on the alternation of this dynamic nature of condensates over time or under specific cellular contexts (Lyon et al., 2021). For example, during pathological aggregation or stress conditions, these molecules are shown to lose their dynamicity to form immobile states. Often these arrested assemblies have been termed gel- or solid-like, a rheological phenomenon which needs to be explored further (Alberti and Hyman, 2021; Visser et al., 2024). Abnormal phase separation has been speculated to be involved in protein condensation and aggregation, as well as in the development and progression of numerous diseases. Over the past decade, research from various groups has attempted to establish a link between protein phase separation and aggregation, which is relevant in understanding the mechanism of pathogenesis of neurodegenerative disorders (Han et al., 2024; Mathieu et al., 2020; Vendruscolo and Fuxreiter, 2022). While these findings have deepened our mechanistic understanding of such diseases, they also highlight the need to explore the complex nature of protein assemblies for appropriate therapeutic intervention. The current experimental efforts in the fields of phase separation and aggregation are extensively reviewed in the literature (Visser et al., 2024; Hribar-Lee and Luksic, 2024; Chakraborty and Zweckstetter, 2023).

Here, we first sought to define the biophysical characterization and the underlying principles of the protein assemblies. We further highlight the studies that demonstrate the functional relevance of different protein assemblies that have been under study for their condensate-forming and aggregation potential. In this review, we aim to enhance the current understanding of the connection between protein phase separation, aggregation, and major aggregation-linked diseases, and predict future advancements needed for establishing a mechanistic foundation across cellular pathways governed by condensates and protein aggregates.

## 2 Assembly of biomolecules in cells

**FIGURE 1:**
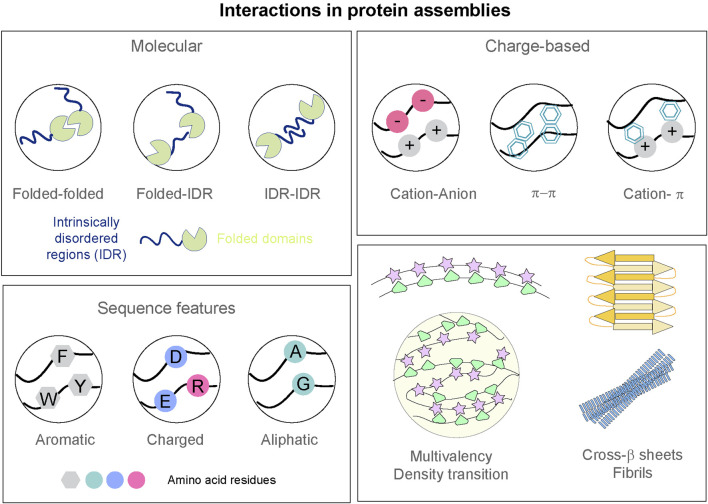
Interactions governing formation of protein assemblies. *Top left:* Interactions between folded domains and intrinsically disordered regions (IDR) of protein polypeptides. *Top right:* Schematic representation of the multivalent interactions—including electrostatic, π–π stacking, cation-π, that can possibly drive the assembly of proteins. *Bottom left:* Amino acid residues with aromatic, charged, and aliphatic natures can dictate the sequence features of the protein polypeptides. *Bottom right:* Multivalency in biomolecules can lead to condensate formation. Cross-β architecture and ordering can lead to more rigid and irreversible amyloid states.

Biomolecules form hierarchies of assemblies depending on the governing interactions and the underlying grammar of amino acid sequences (Figure 1). Biophysical properties of the protein assemblies can contribute to the formation, stability, and ultimately functional relevance in the context of a cell. We further describe some of these assemblies based on their measurable and predictable properties.

### 2.1 Condensates

Biological systems have recently garnered attention for their ability to form dynamic liquid-like compartments through the multivalent interactions of proteins and/or nucleic acids, which allows them to organize cellular biochemistry without membranes (Banani et al., 2017). Weak, multivalent, and reversible interactions among intrinsically disordered regions (IDRs), low-complexity domains, and folded domains of the proteins have been speculated to be involved in the formation of these dynamic, membrane-less organelles (Figure 1, Types of multivalent assemblies). The classification of these assemblies, the nomenclature (called as dynamic droplets/condensates/hubs/puncta) and the underlying principles have been a topic of debate. The nomenclature is ambiguous due to lack of concrete biophysical characterization of these membraneless assemblies but for simplicity and clarity, we will use the term “condensates” throughout the review. These condensates are shown to undergo coalescence with high internal molecular mobility in several cases (Choi et al., 2020; Feng et al., 2019). The functional role of condensates in organizing biochemical reactions has been elucidated in biological processes linked to nucleoli, stress granules, and P bodies (Boeynaems et al., 2018). Additionally, cellular processes, like transcription (Pei et al., 2025), translation (Ramat and Simonelig, 2022), and DNA damage repair (Kilic et al., 2019; Pessina et al., 2019), are under scrutiny for studying the regulatory control of biomolecular condensates.

Various groups have investigated the dynamics, as well as the viscoelasticity and surface tension, of condensates involved in different cellular processes. The condensate assemblies are highly responsive to environmental changes like concentration of salt, pH, temperature, crowding, and stress. Aberrant formation of condensates due to the deregulation of cellular processes has been hypothesized (Alberti and Hyman, 2021; Mathieu et al., 2020). Aging and maturation of condensates leading to loss of dynamicity have been linked to neurodegenerative diseases such as Alzheimer’s disease and Parkinson’s disease (Alberti and Hyman, 2021; Visser et al., 2024; Han et al., 2024; Mathieu et al., 2020; Ray et al., 2020).

Furthermore, percolated networks can arise with a mixture of individual biomolecules, typically proteins or nucleic acids. In a percolated network, the multivalent biomolecules behave like associative polymers that form reversible, noncovalent cross-links. When the number of cross-links crosses a threshold known as the percolation threshold, the molecules are incorporated into a system-spanning (percolated) network. The cross-linking in such percolation networks is formed via transient or stable non-covalent interactions, often driven by multivalent domains (e.g., repeats of SH3, PRM, or intrinsically disordered regions). Mittag and Pappu (2022) offer a comprehensive “phase separation coupled to percolation” (PSCP) framework that unifies phase separation and network formation in biomolecular condensates (Mittag and Pappu, 2022). The cellular condensates are dense liquid droplets with a viscoelastic network whose properties derive from the interplay between polymer-like networking (percolation) and density-driven phase separation. This coupling can lead to sequence- and topology-specific internal structures and allows the formation of protein clusters even below phase separation thresholds (Mittag and Pappu, 2022). Further, Das and Deniz (2023) focus on how topological characteristics, particularly percolation and polymer entanglement, shape condensate architecture and material behaviours. The network topology provides a mechanistic basis for condensates’ connectivity, while entanglement influences their dynamics and viscoelasticity, highlighting open questions for future research (Das and Deniz, 2023). Deniz (2022) extends the conceptual discussion by linking percolation theories from polymer physics to biomolecular condensation phenomena observed experimentally and computationally (Deniz, 2022). This supports the prediction that pre-percolation clusters can form well below saturation concentrations, challenging classical nucleation models and implying a role for percolation in diseases like neurodegeneration. This line of thought supports the integrated perspective that condensate behaviours emerge from a balance of density transitions and network connectivity. The percolated networks may form within biomolecular condensates and can impart biophysical features to the assemblies. These networks may be subjected to changes depending on the proteins involved and environmental factors. Hence, these biophysical features have to be thoroughly investigated to understand and predict the protein behaviour in cells.

### 2.2 Reversible fibrils

Reversible fibrils are filamentous assemblies that can dynamically assemble and disassemble under physiological conditions, often serving as regulatory or storage structures (Saad et al., 2017). The fibrils are ordered and β–sheet–rich but may not be tightly packed as amyloid fibrils. The reversibility of the fibrils is attributed to the changes in conditions such as pH or phosphorylation. Several of these reversible amyloids have been demonstrated to work in signaling, scaffolding, or reversible sequestration in the cellular *milieu* (Saad et al., 2017; Gui et al., 2019; Lacroix et al., 2021). Proteins like hnRNPA1 have been predicted to contain low-complexity domains that facilitate reversible fibril formation (Gui et al., 2019). These domains can form labile, reversible fibrils that are sensitive to environmental conditions, aiding in dynamic cellular processes. Several peptide hormones, including insulin, somatostatin, growth hormone, and β-endorphin, are known to adopt reversible amyloid-like states as part of their normal physiological packaging and storage (Horváth et al., 2023; Maji et al., 2009; Anoop et al., 2014; Jacob et al., 2016). In the secretory granules of endocrine and neuroendocrine cells, these hormones form dense, amyloid-like aggregates that serve to concentrate and stabilize hormones for regulated secretion. Unlike disease-associated amyloids, these aggregates are dynamically reversible: they disassemble upon exocytosis to release active, functional monomers in response to stimuli (Maji et al., 2009). This paradigm of reversible amyloid formation illustrates how nature harnesses protein assemblies and conformations with amyloid-like structures for beneficial roles, distinguishing them from pathogenic aggregates in neurodegenerative diseases.

### 2.3 Amyloid fibrils

Amyloid fibrils are highly ordered, β–sheet–rich polypeptide aggregates characterized by a cross-β spine architecture, where β-strands are stacked perpendicularly to the fibril axis (Maji et al., 2009; Toyama and Weissman, 2011). These structures are extremely stable, resistant to proteolysis, detergents, and heat, and exhibit very low molecular mobility in cells. Amyloid fibrils grow by recruiting monomeric proteins, and can seed the formation of new fibrils in a prion-like manner. The nucleation step is crucial to the assembly of amyloid aggregates and is often thought to be the rate-limiting step in the assembly process (Rambaran and Serpell, 2008). Pathological amyloids, such as Aβ in Alzheimer’s (Selkoe, 1991), α-synuclein in Parkinson’s (Goedert et al., 2001), and TDP-43 (Prasad et al., 2019) or FUS in ALS are implicated in neurodegenerative diseases (Takanashi and Yamaguchi, 2014).

A critical determinant of function *versus* pathological state of proteins may be their way of assembly into higher-order assemblies and, therefore, the emergent properties of these assemblies. Together, the elucidation of molecular aspects of protein assemblies marks an important paradigm shift in viewing protein assemblies through the dual lenses of biological assemblies and network physics, and calls for further exploration of their topology-function relationships in health and disease.

The above classified assemblies and their properties have been explored in detail cellular processes and in the context of disease. Hereafter, we will delve into the studies that highlight the interplay between protein phase separation and aggregation as observed in various proteins to date (Table 1).

**TABLE 1:** Protein aggregation and condensation in physiological contexts.

	Observations		Key references
Protein	Aggregation	Phase separation	
Stress granules			
G3BP1	G3BP1 drives the nucleation stress of granule formation, aiding in the formation and dynamics of protein-RNA aggregates	G3BP1 undergoes phase separation with RNA to form condensates (possibly leading to stress granule formation) in response to cellular stress G3BP1 condensation is regulated by protein concentration, RNA binding, and interactions with partners like Caprin-1 and USP10	Matsuki et al. (2013), Guillén-Boixet et al. (2020), Yang et al. (2020), Parker et al. (2025), Schulte et al. (2023)
hnRNPA1	Mutations in hnRNPA1 promote irreversible amyloid formation and impair droplet dynamics, potentially leading to pathological protein aggregation Dysregulated PARylation contributes to hnRNPA1 aggregation and neurodegenerative disease	hnRNPA1 undergoes phase separation via its low-complexity domain, which aids in stress granule assembly during cellular stress	Molliex et al. (2015), McGurk et al. (2018), Linsenmeier et al. (2023), Das et al. (2025)
TDP-43	When TDP-43 exceeds a threshold and RNA recognition motif 1 (RRM1) unfolds, exposed hydrophobic regions and cysteines form disulfide bonds, leading to amyloid-like aggregates in stress granules Mutations that prevent TDP-43 demixing block pathological aggregation, showing that stress granules can become pathological when intra-condensate organization seeds facilitate the aggregation process	TDP-43 forms condensate within stress granules under cellular stress, with intra-condensate demixing modulated by its multidomain structure and RNA interactions YAP directly binds to TDP-43 and regulates the dynamic behavior of stress granules and condensates by promoting TDP-43 homotypic multimerization and phase separation This interaction helps maintain the number and fluidity of TDP-43 condensates while preventing their pathological solidification under stress conditions	de Boer et al. (2020), Yan et al. (2024), Scherer et al. (2024), Matsushita et al. (2025)
Secretory granules			
Insulin	At insulin injection sites, insulin can aggregate into amyloid fibrils, leading to localized amyloidosis; these fibrils are stabilized by hydrophobic interactions and disulfide bonds Insulin aggregation is influenced by pH, temperature, and reducing agents, while macrocyclic compounds like pentaphenarene (PPAS) can inhibit and reverse fibrillation	Insulin undergoes phase separation and forms condensates for efficient storage in pancreatic β-cell secretory granules, a process facilitated by chromogranin proteins Insulin can assemble into metastable nano-condensates that serve as precursors to aggregation and fibrillation	Parchure et al. (2022), Vorontsova et al. (2015), Toledo et al. (2023), Suladze et al. (2024), Karmakar et al. (2022), Wang et al. (2014)
Chromogranin	Chromogranin condensates transition from a liquid-like to a solid aggregate state, hindering the protein’s ability to recruit hormones, disrupting proper packaging Premature aggregation in the TGN hinders hormone storage and secretion	Chromogranin A and B undergo phase separation in the trans-Golgi network, forming condensates that act as scaffolds for hormone packaging into secretory granules	Parchure et al. (2022), Campelo et al. (2024)
Protein assemblies in diabetes			
IAPP	In type 2 diabetes, IAPP aggregates into highly stable, β-sheet-rich amyloid fibrils within the islets, contributing to β-cell dysfunction and death	IAPP is stored in pancreatic β-cell secretory granules in a soluble or reversible condensate state It prevents premature aggregation and allows for regulated hormone secretion	Westermark and Westermark (2011), Akter et al. (2016), Mukherjee et al. (2017), Wang et al. (2014), Hassan et al. (2022)
Proinsulin	Misfolded proinsulin forms insoluble aggregates in the ER, leading to β-cell dysfunction and diabetes Genetic mutations or ER stress can increase proinsulin aggregation, as in MIDY, where mutant proinsulin traps wild-type proinsulin and reduces insulin production	Proinsulin forms dynamic, reversible condensates during maturation, essential for proper insulin sorting and secretion ER chaperones prevent unwanted condensation, but in the Golgi, condensation enables efficient hormone storage; disruption can cause harmful aggregation and β-cell failure	Parchure et al. (2022), Toledo et al. (2023), Arunagiri et al. (2019), Parashar et al. (2021), Arunagiri et al. (2024)
Neurodegeneration-associated proteins			
Tau	Tau droplets serve as intermediates in forming Tau aggregates by spatial organization within the cytoplasm Also, it helps in forming amyloid aggregates by being involved in the formation of toxic oligomers that develop into amyloid aggregates	Tau undergoes phase separation to form Tau condensates in cells Tau condensates regulate microtubule assembly but have been involved in the formation of toxic oligomers that develop into amyloid aggregates	Wegmann et al. (2018), Ambadipudi et al. (2017), Hernández-Vega et al. (2017), Tsoi et al. (2025)
Amyloid-β (Aβ)	The condensates mature into aggregates via the biomolecular condensate phase of the peptide, which increases the fundamental nucleation step in Aβ42 aggregation These aggregates are involved in neurodegenerative diseases such as Alzheimer’s disease and other amyloid diseases	Amyloid-β also forms condensates primarily of Aβ42 with the help of lipids as a cofactor. Thus, it is present near or on the cell surface Also, it needs a cell to grow into aggregates, supporting the role of the cell membrane in the development of the condensates	Chen et al. (2017), Yang et al. (2019), Morris et al. (2024), Šneiderienė et al. (2025)
α-Synuclein (αSYN)	The condensates formed help in an increased rate of amyloid aggregates The fibril aggregates formed from the condensates are seen in neurodegenerative diseases like Parkinson’s disease	α-Synuclein also forms condensates by phase separation These condensates help in fibril aggregation through C-terminal domains and electrostatic forces α-Synuclein condensate formation may be implicated in driving disease associated aggregation of the protein	Luk et al. (2009), Sacino et al. (2014), Ray et al. (2020), Chandran et al. (2025), Hardenberg et al. (2021)

This table summarizes the aggregation and condensation behaviours of various proteins, highlighting how these behaviours are influenced by different physiological conditions.

## 3 Implications of condensate formation and aggregation in physiology and disease

### 3.1 Stress granules

Stress granules (SGs) are membrane-less cytoplasmic assemblies of mRNA and proteins that form in response to cellular stress (e.g., oxidative stress, heat shock, viral infection) (Alberti and Hyman, 2021). They are known to sequester stalled translation initiation complexes, helping to reprogram gene expression and protect mRNA until stress resolves (Ramaswami et al., 2013). Recent evidence indicates that the formation of stress-granules is attributed to multivalent interactions between involved proteins (Riback et al., 2017). Furthermore, SGs have been increasingly linked to neurodegenerative diseases because alteration of their phase-behaviour of the proteins can lead to persistent, aggregation-prone assemblies. We summarize below the key reports on proteins involved in the formation of stress granule assemblies.

#### 3.1.1 G3BP1

G3BP1 and G3BP2 proteins are shown to assemble stress granules (SGs) through multivalent crosslinking of ribonucleoprotein particles (RNPs) (Matsuki et al., 2013; Guillén-Boixet et al., 2020). The protein G3BP1 serves as a central molecular switch controlling stress granule formation via phase separation (Yang et al., 2020). G3BP1 promotes interaction with RNA at the intermolecular level (Parker et al., 2025) to initiate RNA-dependent condensate formation, potentially driving stress granule assembly. It is speculated that extrinsic binding factors/proteins can modulate this process, either promoting or inhibiting stress granule assembly by interacting with the G3BP1-driven core network. G3BP proteins, thus, act as a central node and molecular switch in SG assembly (Guillén-Boixet et al., 2020). The G3BP1 homodimer undertakes both distinct expanded and compact conformations in solution, with transitions modulated little by physiological changes in ionic strength and pH, as G3BP1 can undergo RNA-independent phase separation at pH 6. G3BP1’s function is modulated by interactions with proteins such as Caprin-1 and USP10. Caprin-1 promotes SG formation, while USP10 inhibits it. Interestingly, the C-terminal domain of Caprin-1 can undergo spontaneous phase separation, facilitating SG assembly, whereas its N-terminal domain suppresses G3BP1’s phase separation, highlighting a regulatory mechanism (Schulte et al., 2023; Han et al., 2025).

More importantly, reconstituted stress granules from cell lysates show that G3BP1 concentration and RNA availability determine whether assemblies remain liquid-like or undergo aggregation-prone maturation (Freibaum et al., 2021). It was demonstrated that G3BP1 also has “condensate chaperone” functions, which enhance the assembly of SGs but get dispersed after the initial condensation. After the granule formation, G3BP1 is used for the RNA component of granules to continue *in vitro* and in cells when RNA decondensers are inactivated. These findings demonstrate that G3BP1 functions as an “RNA condenser” (Parker et al., 2025). The evidence also showcases the role of G3BP1 conformational plasticity in condensate assembly and aggregation. Thus, the evidences suggest that the critical interplay of interactions in proteins and RNA can dictate the properties and the fate of the assemblies formed. The role of RNA (its sequence and/or its length in affecting the network formation and maturation of protein assemblies. Hence, a detailed study of this relationship is essential to distinguish properties of reversible condensate formation of G3BP1 from pathological aggregation and to understand their impact on cellular stress responses.

#### 3.1.2 hnRNPA1

Heterogeneous nuclear ribonucleoprotein A1 (hnRNPA1) is one of the most abundant proteins expressed in eukaryotic cells and is a pivotal RNA-binding protein involved in RNA metabolism processes such as pre-mRNA splicing, transcriptional regulation, localization, and transport (Guil et al., 2006). Under cellular stress, hnRNPA1 contributes to the formation of stress granules and phase separation, a process driven primarily by its intrinsically disordered low-complexity domain (LCD) (Molliex et al., 2015; Wang et al., 2021). Reversible interactions within hnRNPA1 droplets decrease their dynamicity, reinforcing phase separation and possibly facilitating stress granule assembly (Molliex et al., 2015; Wang et al., 2021). Mutations promoting irreversible amyloid formation impair droplet dynamics, potentially leading to pathological aggregates (Molliex et al., 2015). Poly (ADP-ribosyl)ation (PARylation) of hnRNPA1 enhances its phase separation and promotes co-phase separation with other RNA-binding proteins like TDP-43 (McGurk et al., 2018). Additionally, increasing cellular PARylation levels by knocking down PARG delays the disassembly of stress granules, which contain components like hnRNPA1 and FUS (Liu et al., 2025). This modification regulates stress granule dynamics and, when dysregulated, may contribute to neurodegenerative disease pathogenesis. For establishing a link between phase separation and aggregation of hnRNPA1, Linsenmeier et al. showed that the hnRNPA1 low-complexity domain forms amyloid fibrils predominantly at the condensate interface, suggesting that molecular orientation and conformation at the boundary actively promote fibrillation (Linsenmeier et al., 2023). In contrast, Das et al. reported that condensates formed by prion-like domains, while also nucleating fibrils at their interfaces, inhibit fibril growth within and act as protein sinks, sequestering soluble proteins and releasing them slowly, thereby restricting fibril development in the surrounding phase (Das et al., 2025). These reports underline the importance of understanding the emergent properties of condensates and their interfaces. The systems used to study the biophysical properties can also influence the pathogenic aggregation pathways and therefore have to be systematically characterized with caution.

#### 3.1.3 TDP-43

TDP-43, a nuclear RNA-binding protein linked to amyotrophic lateral sclerosis (ALS) and frontotemporal dementia (FTD), is known to mislocalize and accumulate in cytoplasmic stress granules under cellular stress (de Boer et al., 2020). Under oxidative conditions, TDP-43 undergoes intra-condensate demixing (Yan et al., 2024). TDP-43 forms a sub-phase inside the granule that acts as a seed for pathological amyloid-like aggregates (Yan et al., 2024). TDP-43-polyA interactions are formed when TDP-43 binds with RNA (Scherer et al., 2024), which further can alter the viscoelastic properties of the condensates. The presence of polyA increases elasticity, making viscosity and elasticity comparable in magnitude. These findings suggest that the multidomain structure of TDP-43 and its RNA interactions orchestrates condensate organization and modulates their viscoelastic properties (Matsushita et al., 2025). The study highlights how stress granules, normally protective, can become pathological environments when intra-condensate organization facilitates protein aggregation (Matsushita et al., 2025). Fernandes et al. also demonstrate that while stress granules can facilitate TDP-43 aggregation, they are not strictly required for its cytoplasmic assembly (Fernandes et al., 2020). In yeast, most TDP-43 foci initially co-localize with SG-like structures, but many later dissociate and associate with P-bodies or exist independently. Similarly, in mammalian cells, disrupting SG formation reduces but does not abolish TDP-43 foci, highlighting a modulatory rather than essential role for SGs (Fernandes et al., 2020). These observations reflect how sequence composition, RNA concentration, and composition of biomolecules can tune the internal dynamics and material states of condensates. Therefore, such emergent viscoelastic behaviour can help understand the underlying transition from functional RNA–protein assemblies to aberrant, aggregation-prone states linked to disease.

Overall, the examples of proteins from stress granule assemblies demonstrate that the state and composition of protein assemblies can be influenced by intrinsically disordered regions, post-translational modifications, and environmental cues. The formation and maintenance of SGs is a vital phenomenon to demonstrate the complex balance between condensate formation and amyloid aggregation of proteins. Understanding the inherent tug-of-war identifies molecular targets to restore stress granule dynamics and design therapeutics for neurodegeneration and stress-related diseases. Future research in this area can be directed at understanding the changes associated with the dynamicity of molecules, material properties, and rheology of heterogeneous systems.

### 3.2 Secretory granules

#### 3.2.1 Insulin

Insulin, a peptide hormone vital for glucose regulation, has a notable tendency to undergo aggregation under specific conditions (Mori et al., 2022). This behaviour holds both physiological importance, as seen in insulin’s storage within pancreatic β-cell secretory granules, and pathological relevance, such as the formation of amyloid deposits at injection sites for diabetic patients. In pancreatic β-cells, insulin is stored in secretory granules, with studies suggesting that chromogranin proteins facilitate this by undergoing condensate formation in the trans-Golgi network, recruiting insulin for packaging without needing specific cargo receptors (Parchure et al., 2022). Insulin can also form metastable nano-condensates, or “mesoscopic clusters”, which are precursors to fibrillation (Vorontsova et al., 2015; Toledo et al., 2023). Pathologically, at frequent insulin injection sites, insulin can aggregate into amyloid fibrils, leading to localized amyloidosis, where the fibrils arestabilized by hydrophobic interactions and disulfide bonds, forming the amyloid core (Suladze et al., 2024). Factors like pH, temperature, and reducing agents significantly influence the aggregation; for instance, protein disulfide isomerase can catalyse disulfide bond reduction, promoting structures rich in antiparallel β-sheets (Suladze et al., 2024). Understanding these mechanisms and the connection between phase separation and aggregation is crucial for improving insulin storage and delivery as well as for mitigating amyloid-related complications in diabetic patients.

Recent research has significantly advanced our understanding of the intricate processes governing insulin aggregation and condensation *in vitro*, revealing diverse pathways, intermediate states, and potential applications. Across separate investigations, scientists have illuminated how molecular design, environmental factors, and specific interacting agents influence the formation and characteristics of insulin aggregates, from amyloid fibrils to reversible condensates. A recent study showed that the length of an oligolysine segment (Kn) from insulin-derived peptides (ACC1-13Kn) determines their ATP-driven aggregation (Dec et al., 2023). The shorter Kn variants (n = 8, 16) formed amyloid fibrils directly, whereas longer ones (n = 24, 32, 40) first underwent phase separation before forming insulin fibrils. ATP acted as a key counterion, likely engaging the cationic oligolysine via Coulombic attraction, thereby directing these transitions. Given ATP’s role in secretory granules, this mechanism may underlie its influence on insulin storage and aggregation. Environmental factors such as ionic strength and pressure further modulated these phase-to-fibril conversions (Dec et al., 2023). Expanding on the concept of varied aggregation pathways, another study meticulously differentiated two distinct types of human insulin amyloids: (i)-amyloid and (r)-amyloid. The (i)-amyloid, formed without a reducing agent, characteristically exhibited a transient phase separation phase, manifesting as fluid, hydrophobic droplets that subsequently matured into fibrils. Its formation was notably inhibited by 1,6-hexanediol, which dissolved these droplets and, importantly, preserved cytotoxic soluble oligomers. This pathway was also accelerated by chaotropic anions. In stark contrast, the (r)-amyloid, formed in the presence of a reducing agent, directly generated amorphous aggregates without any observable phase separation intermediate. These aggregates displayed lower fluidity, stronger hydrophobic interactions, and were promoted by kosmotropic ions, underscoring fundamental differences in their formation mechanisms and potential for toxicity (Mori et al., 2022). Further demonstrating the versatility of insulin’s self-assembly, Karmakar et al. (2022) explored zinc-induced insulin condensation (Karmakar et al., 2022). This research identified an optimal equimolar ratio of zinc to insulin combined with thermal stress that promoted robust insulin aggregation into a unique protein condensate. This process involved a shift in insulin’s isoelectric point and the formation of specific conformational variants (Karmakar et al., 2022). The resulting condensates exhibited hallmark properties of protein condensates: a porous, amorphous, and macromolecular structure, viscoelastic behavior, and remarkable reversibility, capable of dissolution upon pH reduction.

Collectively, these studies underscore the complex and tunable nature of insulin aggregation, ranging from ATP- and oligo-lysine-driven phase separation intermediates to distinct amyloid polymorphs and zinc-induced condensates with engineered functionalities. This body of work aligns with the broader understanding that functional amyloids can serve as natural storage mechanisms for peptide hormones (Maji et al., 2009), extending this concept to the intricate behaviors of insulin in physiological contexts.

#### 3.2.2 Chromogranin

Chromogranin proteins, particularly chromogranin A (CgA) and chromogranin B (CgB), are integral to the formation of secretory granules in endocrine and neuroendocrine cells (Feldman and Eiden, 2003). Recent studies have elucidated that these proteins undergo phase separation within the trans-Golgi network (TGN), facilitating the packaging of hormones like insulin into secretory granules (Parchure et al., 2022).

*In vitro* studies have shown that CgA aggregation can be influenced by divalent cations such as zinc and magnesium, as well as an aggregation chaperone [hexahistidine-epitope-tagged secreted alkaline phosphatase (SEAP-His)] (Jain et al., 2002). The presence of calcium ions has also been shown to significantly affect the secondary structure of CgA and to promote an increased rate of CgA aggregation at pH 5.5 compared to pH 7.5. This suggests that granin aggregation depends on conditions relevant to the TGN, namely, a mildly acidic pH (∼5.5–6.1) and millimolar calcium concentrations. Inside neuroendocrine cells, chromogranins themselves aggregate and are packaged within secretory granules through a process of selective aggregation (Jain et al., 2002). Furthermore, they promote the sorting of associated hormones into the regulated secretory pathway by co-packaging them inside dense-core secretory granules. CgA has been demonstrated to play an important catalytic role in secretory granule biogenesis and can act as a modulator for endocrine cell secretory activity (Feldman and Eiden, 2003). Therefore, chromogranin aggregation is a vital step governing the packaging and storage of hormones, neuropeptides, and neurotransmitters within granules in these cells. While the expression of CgA itself is sufficient for secretory granule formation, the precise role of CgA aggregation in secretory granule biogenesis is understudied. Characterizing the nature of CgA aggregates within secretory granules and their role in granule formation and the regulated secretory pathway is essential for a clearer understanding of secretory granulogenesis. This understanding has been advanced by the proposal that phase separation may be the key mechanism for secretory protein sorting (Parchure et al., 2022). Unlike often irreversible and pathological protein aggregation, phase-separated condensates are shown to form dynamic compartments, offering a flexible sorting mechanism that is relevant for the regulation of cellular processes. This study demonstrated that CgB, and to a lesser extent CgA, undergo phase separation *in vitro* at a mildly acidic pH (6.1), mimicking the TGN environment (Parchure et al., 2022). These formed droplets displayed classic liquid-like properties, such as fusion and rapid recovery after photobleaching. Crucially, the acidic pH of the TGN was identified as the primary trigger for CgB phase separation, with high calcium concentrations being dispensable at physiological TGN levels. In contrast, high zinc concentrations led to non-dynamic, solid CgB aggregates, highlighting the distinct nature of phase separation *versus* irreversible aggregation. Within living cells (INS1 832/13 beta-cells and HeLa cells), both proinsulin and CgB formed dynamic, punctate, condensate-like structures at the Golgi apparatus, confirming their liquid-like behavior *in vivo*. Furthermore, CgB condensates effectively recruited proinsulin *in vitro*, and remarkably, also recruited other constitutively secreted proteins like LyzC and Cathepsin D, suggesting that client recruitment is largely non-specific and depends on the cargo’s abundance (Parchure et al., 2022). A critical insight was that only the liquid-like condensates of CgB could recruit clients; solid, zinc-induced CgB aggregates failed to do so, underscoring the paramount importance of the material properties of the assembly for proper sorting (Parchure et al., 2022). The intrinsically disordered N-terminal domain of CgB was identified as the primary driver of its phase separation capability and its ability to induce ectopic granule formation in non-secretory cells. Functionally, CgB-driven phase separation was found to be vital for accurate cargo sorting (e.g., PC2 localization) and the efficient secretion of mature insulin, facilitating granule biogenesis and hormone release (Parchure et al., 2022). In conclusion, this research establishes phase separation of Chromogranin B (CgB) at the trans-Golgi network as a fundamental molecular mechanism for sorting proinsulin and other proteins into secretory granules. CgB condensates act as a dynamic “client sponge,” recruiting proteins based on abundance rather than specific sequences. This dynamic, liquid-like state is essential for precise protein sorting, efficient granule formation, and robust insulin secretion, thereby resolving a long-standing question in pancreatic beta-cell biology regarding proinsulin sorting. Granins, as helper proteins, are themselves able to form aggregates and are stored along with proteins to be secreted within electron-dense core granules inside the cells. The storage and release of peptides/proteins inside the secretory granules thus depend on the kind of aggregates the granins form (Sobota et al., 2006). Therefore, characterizing the nature of chromogranin aggregates within secretory granules remains a crucial area of investigation. Research on secretory granule proteins provides critical insights into the fundamental mechanisms of protein self-assembly and paves the way for future therapeutic strategies.

## 4 Protein aggregation in human diseases

### 4.1 Protein misfolding and aggregation in diabetes

#### 4.1.1 Islet amyloid polypeptide (IAPP)

Islet amyloid polypeptide (IAPP, or amylin) is a major secretory product of the pancreatic β-cells, co-secreted and co-stored with insulin, and plays a physiological role in the regulation of satiety, inhibition of gastric emptying, and suppression of glucagon secretion (Westermark and Westermark, 2011; Alrouji et al., 2023). Under normal conditions, IAPP is stored in secretory granules in a soluble or reversible amyloid-like condensate state, which allows for functional biomolecular dense hormone packing and regulated secretion. This reversible condensate state helps preserve IAPP’s functional structure and prevents premature aggregation (Westermark and Westermark, 2011). At the molecular level, IAPP contains a disulfide bond between residues 2 and 7, which is critical to its correct folding, stability, and function. Disruption of this bond alters IAPP’s aggregation properties, initiating amyloid formation and β-cell death (Akter et al., 2016). In type 2 diabetes, IAPP has a strong tendency to pathologically aggregate into highly stable, β-sheet-rich amyloid fibrils within the islets of Langerhans, a process observed in over 90% of patients with the disease (Westermark and Westermark, 2011; Mukherjee et al., 2017). These amyloid deposits are characterized by a cross-β spine architecture, which can be formed from extended β-strands or, in some cases, β-hairpin motifs. This structure makes them extremely resistant to degradation and effectively irreversible under physiological conditions. The aggregation of IAPP is a nucleation-dependent process, in which monomeric IAPP is recruited to growing fibrils, and can be seeded in a prion-like manner by preformed aggregates (Westermark and Westermark, 2011; Mukherjee et al., 2017). Cross-seeding is a process in which amyloid fibrils of one protein act as a seed for the aggregation of another protein. This phenomenon is implicated in the pathology of several diseases, including Alzheimer’s, Parkinson’s, and type 2 diabetes. This is due to amyloid fibrils sharing significant similarity in both sequence and structure, which enables their β-sheet regions to effectively cross-seed each other’s aggregation (Subedi et al., 2022). Experiments have shown that individuals with type 2 diabetes and Alzheimer’s often exhibit co-localized deposits of IAPP and amyloid-β (Aβ), providing strong evidence of cross-seeding between these amyloid proteins (Subedi et al., 2022). Importantly, cross-seeding can either accelerate or inhibit aggregation depending on the similarity of sequence and structural compatibility (Subedi et al., 2022). Therefore, the accumulation of IAPP aggregates is closely linked to β-cell dysfunction and loss, contributing to the progression of type 2 diabetes by overwhelming the cells’ protein degradation systems, inducing oxidative stress, and ultimately triggering apoptosis (Akter et al., 2016; Miraee-Nedjad et al., 2018).

Insulin normally acts as a potent inhibitor of IAPP aggregation, so insulin resistance leads to increased IAPP production and promotes amyloid formation (Wang et al., 2014). While not all IAPP aggregates are toxic, the formation of specific toxic oligomers and insoluble fibrils is believed to be a critical factor in β-cell death and islet failure. The formation of harmful IAPP aggregates can be further driven by factors such as environmental stressors, genetic mutations, and abnormal post-translational processing (Alrouji et al., 2023). In summary, these findings indicate that the dysfunction of biomolecular condensates initiates IAPP aggregation, which acts as a central mechanism in type 2 diabetes by linking oxidative stress, increased β-cell stress and apoptosis, toxic oligomer formation, proteasome dysfunction, and the promotion of chronic inflammation (Moya-Gudiño et al., 2025).

#### 4.1.2 Proinsulin

Proinsulin is the precursor of insulin synthesized and folded in the endoplasmic reticulum (ER) in pancreatic β-cells. Structurally, proinsulin consists of the A chain, B chain, and C-peptide, which are linked together by disulfide bonds, enabling proper folding and structural stability (Liu et al., 2018). In type 2 diabetes (T2D), pathological aggregation of proinsulin results from disruptions in its trafficking and folding. These disruptions can be triggered by genetic, environmental, or proteostatic stress, forming aggregates (Arunagiri et al., 2019). Proinsulin aggregates can be differentiated from condensates by their stable disulfide complexes, which are slowly cleared by normal cellular mechanisms such as proteasomal degradation (ERAD) or autophagy (ER-phagy) (Cunningham et al., 2019). Notably, the FKBP2, IRE1–XBP1, and PERK/EIF2AK3 pathways in β-cells play crucial roles in regulating chaperone expression and oxidative folding, and their dysfunction is linked to increased proinsulin aggregation (Sowers et al., 2018; Hoefner et al., 2023; Tsuchiya et al., 2018). The impact of these mechanisms is highlighted in monogenic conditions like Mutant INS-gene induced Diabetes of Youth (MIDY), where specific mutations in proinsulin greatly enhance its tendency to form insoluble aggregates. MIDY can be characterized by the accumulation of misfolded mutant proinsulin in the ER of pancreatic β-cells, leading to β-cell failure (Liu et al., 2010). These misfolded mutants lead to the formation of aberrant disulfide-linked aggregates. Moreover, MIDY exerts a dominant-negative effect by isolating wild-type proinsulin within the ER, thereby impairing its export and further diminishing insulin production (Sun et al., 2020).

Besides abnormal disulfide-linked aggregation, proinsulin has a natural tendency to form condensates (Toledo et al., 2023; Campelo et al., 2024). The ER relies on specialized chaperones to suppress unwanted intermolecular condensation, thus facilitating proper intramolecular folding. When proinsulin exits the ER and enters the cis-Golgi, where chaperone activity diminishes and the pH drops slightly below 7.0, its condensation tendency prevails, enabling it to form dimers, hexamers, and higher-order assemblies, a process further facilitated by zinc ions (Toledo et al., 2023). This regulation ensures proinsulin does not prematurely assemble into higher-order structures such as nanocondensates that would disrupt maturation and export through the secretory pathway. The process is further orchestrated by co-condensation with proteins such as RESP18Hh and chromogranin B, which drives the transition of proinsulin from nanocondensates to microcondensates while undergoing phase separation (Toledo et al., 2023). These biomolecular condensates are highly dynamic in contrast to irreversible secretory granules and play an essential role in the sorting, packaging, and budding of immature secretory granules, thereby ensuring efficient insulin storage and secretion (Toledo et al., 2023; Parashar et al., 2021). Studies of the disease-relevant *Akita* mutant proinsulin showed that a dynamic liquid-like condensate accumulates in the ER (Parashar et al., 2021), instead of forming an irreversible aggregate. These condensates can be distinguished from aggregates by their high mobility and the rapid exchange of their contents with the surrounding ER environment. When the ER-phagy receptor pathway is disrupted, *Akita* condensates enlarge and accumulate, especially when the COPII coat subunit SEC24C and the tubular ER-phagy receptor RTN3 are involved. In instances where enlargement is not prevented, condensates can transition into irreversible aggregates, ultimately contributing to β-cell dysfunction and progression of diabetes (Parashar et al., 2021).

It should be noted that proinsulin aggregation (Arunagiri et al., 2019; Westermark and Wilander, 1983) or IAPP aggregation/amyloid formation (Westermark and Wilander, 1983; Hassan et al., 2022) are implicated in the development and progression of the disease itself, directly contributing to β-cell pathology. Insulin aggregation, on the other hand, is primarily an iatrogenic (treatment-induced) or pharmaceutical problem that impacts the effectiveness and safety of insulin therapy, but it is not a part of the underlying biological mechanisms that cause diabetes in the first place. Therefore, while understanding condensate formation and aggregation of these proteins, it is apt to consider the cellular consequences and pathological outcomes.

### 4.2 Neurodegeneration-associated protein aggregation

Neurodegenerative diseases like ALS, FTD, Alzheimer’s, and Parkinson’s have been studied to demonstrate disordered proteins that undergo phase separation to form dynamic biomolecular condensates (Mathieu et al., 2020). The condensates have been postulated to transition into solid, aggregation-prone states, which are linked to disease pathology. This pathological shift, often triggered by mutations, chronic stress, or impaired clearance, leads to amyloid-like inclusions, disrupts cellular homeostasis, and promotes neuronal death. We next discuss the key proteins reported in this context.

#### 4.2.1 Tau

Tau is an intrinsically disordered protein that undergoes condensate formation under physiological conditions, primarily through interactions between its proline-rich and microtubule-binding repeat domains. These interactions are driven by electrostatic forces and π–π stacking, especially in the presence of cofactors such as RNA, heparin, or molecular crowding agents like PEG (Wegmann et al., 2018). Recent reviews in the literature discuss the association of phase separation of Tau with aggregation thereby linking physiology and disease (Li et al., 2023; Rai et al., 2021; Boyko and Surewicz, 2022; Islam et al., 2024).

Tau has been studied extensively to understand the driving forces of condensate formation. One such study focuses on characterizing two mechanistically distinct modes, self-coacervation (SC; Tau–Tau) and complex coacervation (CC; Tau with polyanions such as RNA). The *invitro* experiments complemented with coarse-grained computational modeling show that electrostatics (charge interactions) is the primary driving force for both CC and SC: Tau–RNA and Tau–Tauassociations (Najafi et al., 2021). Droplets formed by complex coacervation (Tau and RNA) are measurably more viscous (higher micro-viscosity) and show greater thermal stability than droplets from Tau self-coacervation (Najafi et al., 2021). These rheological differences imply that distinct internal dynamics and molecular packing in the two types of assemblies, influencing Tau’s conformational ensemble and its subsequent aggregation behaviour.

The formation of dynamic Tau condensates is shown to facilitate spatial organization within the cytoplasm but also predisposes Tau to gelation and amyloid aggregation (Ambadipudi et al., 2017). Over time, these condensates mature into less dynamic, solid-like assemblies, providing nucleation centres for β–sheet–rich amyloid fibrils (Ambadipudi et al., 2017). Also, Tau condensates regulate microtubule assembly but have also been involved in the formation of toxic oligomers that develop into amyloid aggregates implicated in neurodegenerative diseases. Tau condensates can also recruit kinases and RNA, enhancing pathogenic modification and mislocalization, forming a direct bridge from functional phase behaviour to pathological aggregation (Hernández-Vega et al., 2017). Tau showcased condensate formation under cellular conditions, which was predicted to serve as an intermediate on pathway to Tau aggregate formation (Wegmann et al., 2018). Another study, focused on condensate formation and dissolution of wild-type and disease-linked (hyperphosphorylated and missense mutated) Tau variants. All Tau variants [wild-type (WT), hyperphosphorylated (pTau), and missense familial mutation (P301S)] can exist as monomers, nanocondensates, and microcondensates, or a combination of these states (Tsoi et al., 2025). All three forms of Tau exhibited similar condensation formation properties in the presence of increased salt concentration, which showcases that at the nano-condensate level, the forces driving Tau condensation were not significantly affected by changes in charge distribution caused by phosphorylation (Tsoi et al., 2025). A complementary work shows that as Tau oligomerizes the condensate formation propensity declines. The early oligomers can be recruited into condensates where they promote maturation whereas mature fibrils cannot be recruited, implying a temporal window linking condensates to Tau aggregation (Lucas et al., 2025). Thus, these studies highlight the need for exploring types of Tau assemblies and their fate over different time-scales. A recent work by Wen et al. shows that condensate formation and conformational opening are mechanistically coupled, arising from and enforcing a shift to extended Tau conformers that expose the microtubule-binding region, creating locally concentrated, interaction-rich environments that lower nucleation barriers and accelerate amyloid formation of Tau (Wen et al., 2025). Disease-associated mutations (P301L, P301S) further promote conversion from dynamic condensates into irreversible amyloids. Interestingly, in another work, by increasing the fibril nucleation barrier, peptide L-arginine counteracts the age-dependent decline of the Tau condensates by selectively impeding condensate-to-fibril transition without perturbing phase separation in a valence and chemistry-specific manner (Mahendran et al., 2025). This showcases that the small molecule metabolites can enhance the metastability of protein condensates against a condensate-to-amyloid transition, thereby preserving condensate state and function.

Despite strong *invitro* and cellular evidence for the connect between condensates and fibrils, the pathophysiological relevance, precise temporal order of events, and structural transitions within condensates in living neurons remain major gaps in current understanding of Tau pathology.

#### 4.2.2 Amyloid-β (Aβ)

Amyloid-β (Aβ) protein peptides, especially the 42-residue isoform Aβ_42_, are central to Alzheimer’s disease pathology (Finder and Glockshuber, 2007). The peptides are known to accumulate in the brains of diseased patients. Accumulation of Aβ in the brain is considered an early toxic event in Alzheimer’s disease, characterized by the formation of amyloid plaques and neurofibrillary tangles (Chen et al., 2017). The accumulated Aβ fibrils adopt highly ordered, β-sheet-rich structures, with individual peptides stacking into cross-β spines that stabilize the fibril core. Structural studies reveal polymorphic fibril forms, where variations in β-strand arrangement and inter-peptide interactions contribute to morphological diversity and disease-specific aggregation properties (Yang et al., 2019; Kollmer et al., 2021). These Aβ species disrupt cellular homeostasis through oxidative stress, membrane perturbation, and synaptic dysfunction (Musa et al., 2023).

Aβ40 itself can undergo phase separation, forming condensates under physiological-like conditions. Within these condensates, Aβ40 aggregation is accelerated compared to dilute solution, and nucleation occurs preferentially inside the dense phase (Morris et al., 2024). A detailed review which highlights the mechanisms underlying Aβ aggregation and the regulatory role of condensate formation has been published (Niu et al., 2024). Further, Gui, et al. have demonstrated that soluble Aβ oligomers are capable of undergoing phase separation, forming micron-scale, dynamic droplets. Condensate formation accelerates downstream amyloidogenesis, supporting a condensate-mediated microreactor model for primary nucleation (Gui et al., 2023). Further, Aβ peptides can form condensate-like clusters under specific conditions such as acidic pH, metal ion presence (Zn^2+^, Cu^2+^), or membrane interfaces (Šneiderienė et al., 2025). The condensate phase can increase the rate of nucleation for Aβ42 aggregation. The fundamental nucleation step in Aβ42 aggregation can be increased in rate by a biomolecular condensate phase of the peptide. This type of two-stage nucleation process could be a general pathway for neurodegenerative disease-related phase-separating proteins, as seen in other systems such as FUS, TDP-43, α-synuclein, hnRNPA1, and Tau. This evidence suggests that the mechanism of phase separation can govern the pathology of amyloid diseases (Šneiderienė et al., 2025). *In vitro* studies suggest that phase-separated Aβ states can form dynamic oligomeric condensates, especially when combined with polyanions or lipid membranes. These intermediate states lower the energetic barrier for fibril nucleation, potentially representing early-stage pathogenesis in Alzheimer’s disease (Šneiderienė et al., 2025). Although the reversibility of these condensates is limited, their formation and maturation can be influenced by environmental factors such as ionic strength, glycosaminoglycans, and redox states. These findings align with the hypothesis that non-canonical phase separation may serve as a pre-fibrillar organizational mechanism for Aβ. Cumulatively, Aβ condensate formation and associated environmental conditions have key roles in dictating the nucleation, scaffolding and fate of higher order aggregated species implicated in disease pathology.

#### 4.2.3 α-Synuclein (αSYN)

α-Synuclein (αSYN) aggregation is a hallmark of Parkinson’s disease and involves the transition of soluble protein monomers into oligomers, protofibrils, and mature amyloid fibrils (Vidović and Rikalovic, 2022). The aggregation process is driven by misfolding and self-association, often influenced by mutations, post-translational modifications, and/or interactions with membranes (Luk et al., 2009). The resulting amyloid fibrils and oligomeric species are neurotoxic, disrupting cellular homeostasis, impairing synaptic function, and contributing to Lewy body formation in affected neurons (Sacino et al., 2014). αSYN protein contains an amphipathic N-terminal domain, a hydrophobic NAC region (critical for aggregation), and a negatively charged C-terminal tail, which collectively have been speculated to govern its phase separation behavior. *In vitro*, αSYN undergoes phase separation in the presence of molecular crowding agents, RNA, liposomes, or polyphosphates, forming spherical, fusion-capable droplets (Hardenberg et al., 2021). These liquid droplets mature over time into gel-like or solid fibrillar states, especially under stress conditions (e.g., metal ions, low pH, or oxidative stress). Parkinson’s disease mutations (A30P, E46K, A53T) enhance the propensity for condensate formation and show rapid droplet aging, accelerating the transition to fibrils (Ray et al., 2020). Truncation of the acidic C-terminal region, which normally imparts solubility, also promotes phase separation and subsequent aggregation (Ray et al., 2020). These findings suggest that αSYN droplets serve as nucleation-enhancing condensates, linking biophysical phase transitions to disease pathology. The role of electrostatic forces in driving αSYN phase separation is shown by phase diagrams in presence of NaCl (Cui et al., 2025).

A study using cellular models and *in vitro* reconstitution shows that αSYN condensates concentrate monomers and exogenous fibrillar seeds, accelerating seed-dependent fibril elongation and promoting maturation into needle-like, Amytracker-positive aggregates. This study directly correlates condensate formation with propagation of fibrils (Piroska et al., 2023). Surprisingly, another study showcased that αSYN binding with lipids enhances their phase separation, correlating with the Aβ42’s phase separation process (Chandran et al., 2025). Ziaunys et al. show that phase separation of αSYN promotes the formation of structurally diverse fibrils. The presence of condensates accelerates aggregation and produces fibrils with increased morphological variability compared to those formed in bulk solution (Ziaunys et al., 2024). These findings showcase that αSYN exhibits dichotomy associated with the dysregulation of aSYN phase separation, which may directly affect disease pathogenesis. Recent studies also showed that αSYN proceeds through phase separation to increase the rate of amyloid aggregation, β-Synuclein (βSYN), a part of the synuclein family, co-condenses with αSYN and affects its aggregation by negatively regulating the phase separation of αSYN (Xu et al., 2025). βSYN also decreases the mobility of αSYN in αSYN/βSYN coacervates, leading to diminished condensate fusion, growth, and maturation (Xu et al., 2025). These evidences cumulatively suggest the role of βSYN in the neuroprotection of β-Syn and the targeting of α-Syn phase separation in disease treatments (Xu et al., 2025). Hence, this alludes to how heterotypic condensate formation can regulate the fate of complex assemblies and dictate both the nature and extent of aggregation.

The studies highlighted above, using different protein systems and physiological assemblies indicate a complex tug-of-war between the formation of condensates and aggregated fibrils.

## 5 Emergent properties of protein self-assemblies influencing function and disease

**FIGURE 2:**
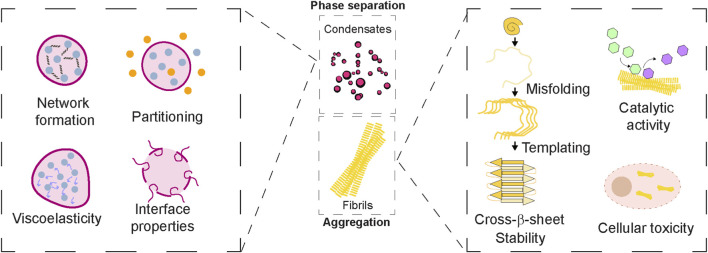
Emergent Properties of Protein Assemblies. *Middle panel:* Protein assemblies such as biomolecular condensates and amyloid fibrils display emergent properties that arise from collective organization beyond individual protein behaviour. *Left panel:* Condensates exhibit dynamic features like network formation, selective compartmentalization/partitioning properties. They can possess material properties in the form of viscoelasticity and contribute to interfacial properties. *Right panel:* Amyloid fibrils are known to acquire structural rigidity via cross-β sheet assemblies as well as templating capacity. While the surface of fibrils can catalyse cellular reactions, the toxic gain-of-function can contribute to cellular toxicity. Together, these emergent properties of protein assemblies underlie both functional roles in physiology and pathogenic outcomes in disease.

The emergent properties that are elucidated from the above studies can assist in guiding future studies in the fields of protein aggregation. For instance, biomolecular condensates display emergent properties that may not be achieved from the sum of their individual molecular components (Visser et al., 2024). Through multivalent interactions, often between intrinsically disordered regions (IDRs) and folded domains, these assemblies undergo phase separation to form dense, droplet-like compartments that concentrate specific biomolecules, creating unique microenvironments where reaction rates, specificity, and selectivity can be dramatically enhanced (Banani et al., 2017; Alberti and Hyman, 2021). These condensates exhibit dynamic behavior, the ability to fuse, deform, and exchange molecules. The assemblies can also mature into viscoelastic or gel-like networks, reflecting material states that are emergent from collective interactions (Elbaum-Garfinkle et al., 2015; Brangwynne et al., 2009; Wang et al., 2022). Crucially, their capacity to tune material properties (viscosity, diffusion barriers) via sequence features, post-translational modifications, and partner interactions enables responsive cellular regulation and biochemical compartmentalization (Wang et al., 2022; Sanfeliu-Cerdán and Krieg, 2025). Such tunability underpins their roles in signalling cascades, stress responses, and metabolic integration. Conversely, dysregulated transitions can drive pathological aggregation linked to neurodegeneration and other diseases (Tejedor et al., 2023). Thus, we highlight the emergent features of protein assemblies (Figure 2) that can link and deconvolute the relation between condensate formation and amyloid aggregation.

### 5.1 Energy landscape in protein assemblies

**FIGURE 3:**
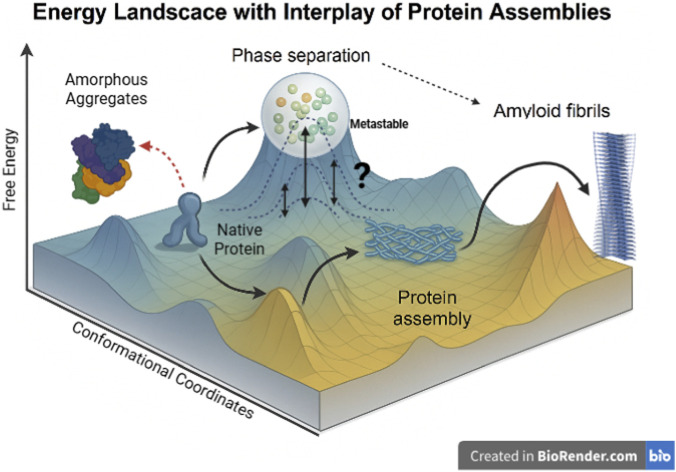
Energy Landscape Depicting the Interplay of Protein Conformational States and Assemblies. This three-dimensional energy landscape illustrates the possible conformational transitions of a protein. The y-axis represents Free Energy, indicating the thermodynamic favourability of each state, with lower energy corresponding to more stable structures. The x- and z-axes collectively represent Conformational Coordinates, which trace the structural transitions influenced by factors such as the protein’s sequence, multivalency, and the cellular environment. The protein starts in a low-energy well as the native protein, from which it can transition along several pathways: It may form ordered protein assemblies in an intermediate energy minimum, or undergo phase separation into dense, droplet-like compartments, which is depicted as a “metastable” state. Alternatively, the native protein can transition to unstructured amorphous aggregates via a high-energy route. The assembled states show potential routes toward the formation of highly stable, misfolded amyloid fibrils, which represent the deepest thermodynamic sink on the landscape. The various connections shown, including the dynamic equilibrium of the phase-separated state and the link between amorphous aggregates and phase separation, highlight the complex interconversion and interplay among these assembly states.

The energy landscape is a conceptual model that maps the free energy of a system as a function of its conformational states. In the context of proteins and their higher-order assemblies, it illustrates the possible assembly forms and their associated stabilities (Figure 3). Condensates formed via phase separation can be driven by multivalent weak interactions (e.g., π-π stacking, cation-π, hydrogen bonding) (Banani et al., 2017). The energy landscape for condensate assemblies has been predicted to dictate dynamic rearrangements of molecules (Biswas and Potoyan, 2025). The assemblies may attain multiple low-energy states, contributing to fluidity and adaptability in the system, and facilitating fast exchange with the surrounding environment (Espinosa et al., 2020). In contrast, amyloid fibrils arise from ordered aggregation, typically involving β-sheet-rich structures (Adamcik and Mezzenga, 2018). The energy landscape here is deep and narrow, featuring high energy barriers between states. The energy landscape also has stable “traps” representing fibril structures (Adamcik and Mezzenga, 2018; Almeida and Brito, 2020). These states may have irreversibility or very slow reversibility with kinetically trapped states.

Due to increasing local concentrations, condensates can lower the effective free-energy barrier for nucleation, biasing ensembles toward aggregation-competent conformers and possibly enabling structural transitions that are improbable in dilute solution. Over time, droplet aging or intra-condensate reorganization can steer the system to thermodynamically favored amyloid minima. A liquid→ gel → solid trajectory driven by accumulation of β-sheet contacts, cross-β spine formation, and stabilizing inter-molecular bonds can be achieved in a temporal manner. Additionally, heterogeneous environments within droplets (interfaces, cofactors, membranes, RNA, post-translational modifications) can create local sub-states and kinetic pathways that favor particular fibril polymorphs, accounting for the structural diversity observed experimentally in case of proteins associated with neurodegeneration. Conversely, chaperones and small molecules can reshape the landscape by stabilizing liquid basins or raising barriers to amyloid basins, thereby decoupling benign condensation from pathological fibrillization. Viewing condensates and fibrils on the energy landscape, thus, can aid to reconcile their dual roles. Hence, understanding the relation of condensates and higher-order assemblies like fibrils in the context of energetics is crucial to map out the processes that are involved in processes like stress granule formation and neurodegenerative diseases.

### 5.2 Viscoelasticity in condensate assembly

Viscoelasticity is the material property describing both viscous (fluid-like) and elastic (solid-like) responses to deformation. Condensates have been shown to exhibit tunable viscoelasticity depending on protein sequence, interactions, and conditions (e.g., salt, pH) (Tejedor et al., 2023). Such assemblies can also behave as viscous liquids or soft solids, characterized by low elastic modulus (G′) and higher loss modulus (G″) in liquid-like states. Percolation within a condensate with the progressive formation of an interconnected molecular network that spans the entire droplet is speculated. As this network strengthens or develops over time, molecular mobility can decrease. The internal rearrangements can slow down leading to increased viscosity and reduced dynamics, attributed to be hallmarks of condensate ageing or maturation. Alshareedah et al. show that condensates of prion-like low-complexity domains of hnRNPA1 behave as aging viscoelastic (Maxwell-like) fluids whose elastic and viscous moduli, and the timescales over which elasticity dominates, are encoded by sequence-specific sticker–sticker interactions (especially aromatic contacts). Aging is shown to be accompanied by disorder-to-order transitions in some protein sequences, producing non-fibrillar, β-sheet–containing semi-crystalline solids (Kelvin–Voigt–like-behavior) (Alshareedah et al., 2024).

The condensates have been claimed to undergo time-dependent aging or hardening into more solid-like states (e.g., gelation or maturation) (Dabbaghi et al., 2021). The molecules in the dense phase can undergo dynamic internal rearrangement, which can influence networking properties while leading to recovery after stress. Fibrils form rigid, elastic structures that exhibit strongly elastic mechanical behavior, characterized by a high storage modulus (G′) and negligible loss modulus (G″), indicating minimal energy dissipation (Dabbaghi et al., 2021). This mechanical profile confers significant resilience and resistance to deformation, allowing fibrils to act as durable structural scaffolds. In pathological contexts, such as Alzheimer’s disease, amyloid fibrils provide a stable yet detrimental framework that contributes to neurodegeneration. Conversely, in functional contexts like spider silk or certain microbial matrices, fibrils perform essential mechanical roles, demonstrating how similar structural properties can underpin both disease and physiological utility (Qi et al., 2023). Thus, percolation can provide the physical basis for the transition from a liquid-like, reversible condensate to a more gel-like or solidified state associated with functional stabilization or pathological aggregation. Taken together, these directions emphasize moving from descriptive accounts of condensate ageing to mechanistic, quantitative maps that connect sequence and composition → percolation/network topology → viscoelastic spectra → functional or pathological outcomes.

### 5.3 Distinct chemical microenvironment

Condensates and fibrils create distinct chemical microenvironments that critically influence the behavior of associated molecules. In condensates, the microenvironment is highly dynamic and selectively permeable, enriched with co-aggregators, RNA, intrinsically disordered proteins (IDPs), and molecular chaperones that modulate assembly and disassembly (Niu et al., 2023). These environments favor weak, multivalent interactions, allowing for rapid exchange with the surrounding cytoplasm and facilitating regulated biochemical reactions. Chaperones, such as HSP70 or HSP40, often localize to condensates to prevent aberrant aggregation, maintaining their liquid-like properties (Li et al., 2022). In contrast, fibrils form a rigid, dehydrated, and sterically constrained microenvironment, often excluding dynamic co-factors and entrapping proteins in kinetically trapped, β-sheet-rich aggregates (Jia et al., 2020; Akaree et al., 2025). Chaperones are generally less effective once fibrils are fully formed, but may engage early oligomeric intermediates to prevent fibrillization. Thus, the transition from condensate to fibril potentially reflects not only a shift in the material state but also a profound remodelling of the molecular milieu and regulatory potential of the assembly.

### 5.4 Network formation of protein assemblies

In biomolecular condensates, the network formation of protein and/or nucleic acid polymers plays a pivotal role in modulating protein aggregation dynamics. These networks, formed through multivalent interactions, can create a dense, highly interactive environment that can either accelerate or decelerate aggregation depending on the molecular context (Fare et al., 2021). Network formation, therefore, can modulate partitioning and viscoelastic behavior in a temporal manner. On one hand, condensates can concentrate aggregation-prone proteins, increase local concentrations, and promote nucleation events that lead to fibrillization. RNA and other scaffolding molecules can also act as structural templates or seeds that stabilize aggregation-prone conformers (Fare et al., 2021; Li et al., 2022). On the other hand, the same networks can buffer aggregation by maintaining proteins in a dynamic, reversible interaction network, especially in the presence of regulatory factors like chaperones or RNA-binding proteins that disrupt pathological contacts (Akaree et al., 2025). Thus, the polymeric network within condensates could function as a double-edged sword, capable of either fostering or restraining protein aggregation based on the balance of intermolecular interactions and regulatory components.

### 5.5 Interface properties

As evidenced by the studied discussed earlier, phase separation not only serves to compartmentalize constituents but also gives rise to constituent emergent properties by the formation of multivalent interaction networks. The emergent properties of biomolecular condensates are speculated to contribute to specific functions like diffusivity and interfacial tension, and have been studied using interdisciplinary approaches in pan-cellular biomolecular condensates (Jeon et al., 2025). While several lines of evidence suggest that condensates have emergent properties, a concrete extrapolation to in-cell systems has not been achieved. One aspect of condensates that is absent from other higher-order assemblies is the interface. Interface serves as a bridge between the coexisting dense phase and the dilute phase in the two-phase regime. The properties of the interface with respect to the mesoscale organization of molecules have been studied (Farag et al., 2022).

In the context of protein aggregation, contrasting observations have been made for interfacial contributions to fibril formation. Linsenmeier et al. investigate how the low-complexity domain (LCD) of the hnRNPA1 protein, associated with amyotrophic lateral sclerosis (ALS), transitions from liquid-like condensates to amyloid fibrils (Linsenmeier et al., 2023). The research demonstrates that amyloid fibril formation predominantly occurs at the interface of the condensates rather than uniformly throughout. This work predicts that the interface plays a crucial role in promoting fibril formation, possibly due to the orientation and conformation of molecules at the boundary (Linsenmeier et al., 2023). As an example of contrasting behaviours, Das et al. recently demonstrated that condensates formed by prion-like domains prone to fibril formation can facilitate nucleation of fibrils at their interfaces while inhibiting fibril growth within their interiors (Das et al., 2025). The fibrils develop in the surrounding dilute phase, and the metastable condensates hinder their growth by sequestering soluble proteins and releasing them slowly, thereby acting as protein sinks.

The emergent properties studied so far have illuminated the intricate interplay between protein phase separation and the aggregation of biomolecular condensates. We further provide a comprehensive overview of conclusions across the various systems studied to provide new directions in the mechanistic understanding of fundamentals of human diseases and potentially the development of targeted therapeutic interventions.

## 6 Conclusion

The interplay between reversible phase separation and irreversible aggregation of proteins is central to understanding both normal cell physiology and disease pathology (Vendruscolo and Fuxreiter, 2022). While phase-separated condensates can organize biochemical reactions and promote adaptability, their transition into aggregates under stress or mutation is linked to neurodegeneration, cancer, and aging (Visser et al., 2024).

Biomolecular condensates form through interactions that often involve IDRs or modular interaction domains that enable flexible and reversible binding. IDRs facilitate π–π stacking, cation-π interactions, electrostatic complementarity, and transient hydrogen bonding, enabling dynamic assembly of biomolecular condensates (Banani et al., 2017). Proteins such as FUS, TDP-43, hnRNPA1, and Tau exemplify this, forming condensates in response to changes in concentration, ionic strength, or temperature. Importantly, RNA molecules can act as scaffolds or regulators of phase separation by stabilizing or disrupting condensates depending on their concentration and sequence specificity (Boeynaems et al., 2018; Boeynaems et al., 2019; Maharana et al., 2018).

While condensates are initially dynamic, exhibiting fusion, internal mobility, and fluorescence recovery after photobleaching (FRAP), many proteins have been reported to undergo aging or maturation over time with loss of dynamicity. This process has been predicted by increased cross-linking, β-sheet formation, and the eventual development of amyloid fibrils, often pertinent in neurodegenerative diseases (Han et al., 2024; Mathieu et al., 2020). Proteins like Tau, α-Synuclein, and FUS exhibit liquid-to-solid transitions influenced by post-translational modifications, mutations, and environmental stressors (Farina et al., 2021). These matured condensates show reduced dynamics and increased structural order, resembling pathological aggregates such as those in Alzheimer’s, Parkinson’s, and ALS. The transition is driven by nucleation-limited kinetics, where the condensate environment accelerates monomer recruitment and fibrillization. On the other hand, ATP-dependent chaperones and RNA helicases modulate condensate dynamics, preventing unwanted aggregation and maintaining cellular proteostasis (Patel et al., 2015; Patel et al., 2017). The transitions of material properties of secretory granules proteins are unexplored in detail. Hence, the need of the hour is to critically evaluate the changes in the material properties of protein assemblies during the cellular processes to assess their importance. While in several studies, FRAP is widely used to probe condensate dynamics, it has major limitations in reporting material properties of the assemblies. FRAP recovery curves are highly model-dependent and conflate diffusion with binding kinetics, making them unreliable as sole evidence for “liquid” behaviour. Partitioning at condensate interfaces, photobleaching artifacts, and fluorescent tags can distort results, while spatial averaging hides heterogeneity and material transitions (Taylor et al., 2019). Thus, FRAP should be combined with orthogonal methods like single-particle tracking, FCS, and micro-rheology for robust interpretation of material properties.

**FIGURE 4:**
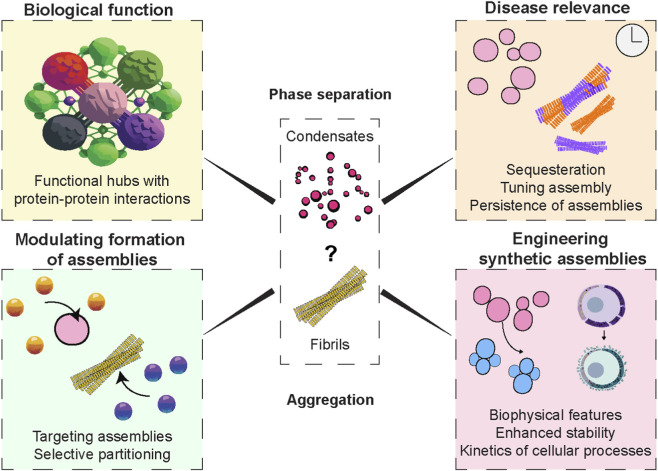
Protein Assemblies in Biology, Disease, and Therapy. Schematic overview of protein assemblies such as condensates and amyloid fibrils, highlighting their dual nature. In physiology, they enable biological functions including compartmentalization, signalling, and structural support. In pathology, aberrant assemblies contribute to diseases such as neurodegeneration and cancer by sequestration and persistence of insoluble aggregates. Therapeutic approaches target their formation, dynamics, or clearance to restore cellular balance. Advances in synthetic biology can harness these principles to engineer programmable assemblies with applications in diagnostics, drug delivery, and biomaterials.

We predict that the study of protein assemblies will have a deep impact on several avenues in the upcoming future (Figure 4). The future research in the arena of linking condensate formation to aggregation will need to focus on the following key aspects of protein assemblies:

1. Molecular Determinants: Defining the precise sequence features, post-translational modifications, and RNA interactions that control whether proteins remain in a dynamic state or lead to the formation of aggregates. The material properties like viscoelasticity have to be understood at the basal level and across the temporal scales to connect the maturation process of assemblies to pathology.
2. Interface Biology: Investigating condensate interfaces as critical sites where amyloid formation is postulated to initiate, and exploring strategies to modulate these boundaries to prevent pathological aggregation.
3. Cellular Stress and Environment: Understanding how pH, ionic strength, crowding, or oxidative stress tips the balance from phase separation to aggregation, especially in aging cells.
4. Therapeutic Modulation: Therapeutic intervention possibilities for biomolecular condensates are an emerging and rapidly expanding area of biomedical research, with several promising strategies under investigation. Developing small molecules, peptides, or nucleic acids that stabilize physiologically functional condensates or dissolve/prevent pathological ones, creating new treatments for diseases like ALS, Alzheimer’s, and cancer. This also enables identifying early condensate-to-aggregate transitions as diagnostic markers, enabling intervention before irreversible damage occurs. A step into precision biology would involve engineering condensates with built-in safeguards against aggregation, and tailoring interventions based on individual genetic mutations that affect protein phase behavior.

Linking the biophysical principles of condensate formation and protein aggregation to therapeutic approaches opens new avenues for treating neurodegenerative and proteinopathies-related diseases. Understanding how biomolecular condensates form, mature, and convert into pathological aggregates offers actionable targets for drug design, molecular chaperone modulation, and phase behavior tuning. Hence, the future research focus would aid in decoding the molecular “switches” that decide between functional condensates and toxic aggregates, and in harnessing this knowledge for therapeutic benefit.
